# Childhood Lead Poisoning: Conservative Estimates of the Social and Economic Benefits of Lead Hazard Control

**DOI:** 10.1289/ehp.0800408

**Published:** 2009-03-31

**Authors:** Elise Gould

**Affiliations:** Economic Policy Institute, Washington, DC, USA

**Keywords:** cost-benefit, economics, housing, lead poisoning

## Abstract

**Background:**

This study is a cost–benefit analysis that quantifies the social and economic benefits to household lead paint hazard control compared with the investments needed to minimize exposure to these hazards.

**Objectives:**

This research updates estimates of elevated blood lead levels among a cohort of children ≤ 6 years of age and compiles recent research to determine a range of the costs of lead paint hazard control ($1–$11 billion) and the benefits of reduction attributed to each cohort for health care ($11–$53 billion), lifetime earnings ($165–$233 billion), tax revenue ($25–$35 billion), special education ($30–$146 million), attention deficit–hyperactivity disorder ($267 million), and the direct costs of crime ($1.7 billion).

**Results:**

Each dollar invested in lead paint hazard control results in a return of $17–$221 or a net savings of $181–269 billion.

**Conclusions:**

There are substantial returns to investing in lead hazard control, particularly targeted at early intervention in communities most likely at risk. Given the high societal costs of inaction, lead hazard control appears to be well worth the price.

Lead poisoning is a serious hazard for children and causes significant biological and neurologic damage linked to cognitive and behavioral impairment (Bellinger 2008a, 2008b). The level of lead exposure has fallen dramatically over the past 30 years because the lead content has been reduced in gasoline, household paint, food canning, industrial emissions, water lead, and other sources, and because public health and housing initiatives have targeted the problem. According to the National Health and Nutritional Examination Survey (NHANES), a population survey administered by the Centers for Disease Control and Prevention (CDC), the geometric mean for blood lead levels (BLLs) for children 1–5 years of age fell from 14.9 μg/dL in 1976 to 1.7 μg/dL in 2006 (CDC 2007b). The number of children 1–5 years of age with BLLs at least 10 μg/dL has fallen from an estimated 13.5 million to 174,000 over the same period (NHANES 2003–2006). Although the 1- to 5-year age grouping is useful for comparison over time, I focus on a cohort of children ≤ 6 years of age in which there are an estimated 194,000 children with BLLs at least 10 μg/dL.

Recent research has indicated that significant neurologic damage to children occurs even at very low levels of exposure (Bellinger 2008a, 2008b; Chen et al. 2007; Lanphear et al. 2005). Preventing these levels of exposure in young children will require controlling a significant and persistent cause of lead poisoning: lead paint used in housing before its ban in 1978. Although pre-1950 house paint has the largest concentration of lead-based paint hazards, house paint produced in 1950–1978 also contains substantial lead content. Poor, urban minorities disproportionately reside in housing units containing lead-based paint hazards, creating significant inequity in health and neurologic outcomes by ethnicity and socioeconomic status (CDC 2004). Because the costs of lead paint abatement are nontrivial and the removal must be done on a unit-by-unit basis (rather than imposed at an industry level), there must be substantial commitment to further reduce lead poisoning among vulnerable children.

A growing body of literature has detailed the economic costs and risks of lead poisoning, including several analyses summarizing these costs and setting them against the estimated costs of lead paint hazard control. However, recent research has broadened still the scope of our understanding of the societal costs of lead poisoning. For example, new studies have begun to analyze the correlation of lead poisoning with crime rates and their associated costs, as well as linking early lead exposure to adult-onset health problems. In this article I aim to comprehensively address the costs and benefits of household lead hazard control vis-à-vis new discoveries in the medical, psychological, and economic literature. I focus on children ≤ 6 years of age, because lead exposure is the highest for this age group, and this is the period when lead exposure produces the most significant damage.

In this analysis, I constructed an upper and lower bound on the cost-effectiveness of strategies to reduce lead exposure. The reasoning behind this methodology is that there is no single estimate that accurately reflects either the costs or benefits of lead hazard control. On the costs side, the actual expense of reducing lead paint hazards in affected homes varies with the extent of interventions required. On the benefits side, the number of children with lead exposure ranges from those reported in state child blood lead surveillance data to those determined from weighted estimates of national surveys. Although several factors could make one extreme or another more credible, it is likely that the truth lies within this interval.

## Incidence of Low-Level Childhood Lead Poisoning

Although the attention on lead and children historically has focused on BLLs of ≥ 10 μg/dL, recent evidence suggests that lower levels incur high individual and societal costs. Although community, medical, and environmental interventions have generally been initiated at a BLL of 10 μg/dL, the government has found no level of exposure to lead below which adverse health effect do not occur (CDC 2004). BLLs between 2 and 10 μg/dL have been found to cause persistent cognitive damage (Bellinger 2008a, 2008b; Binns et al. 2007; Lanphear et al. 2005), and children with BLLs in this range are likely to benefit from aggressive intervention. Table 1 compares the composition of children with BLLs between 2 and 10 μg/dL with the demographic patterns of the entire cohort of children ≤ 6 years of age in 2006. Given limited sample sizes in the data, it is inadvisable to independently measure the characteristics of the population with levels > 10 μg/dL.

Of the 27.97 million children ≤ 6 years of age in the United States in 2006 (U.S. Census Bureau 2008), 24.7%, or 6.9 million, have BLLs between 2 and 10 μg/dL (NHANES 2003–2006). Males, Hispanics, African Americans, and children in households below 200% of the federal poverty line are disproportionately more likely to have higher-than-average lead exposures.

## Sources of Lead and Costs of Lead Hazard Control

Although bans on leaded gasoline and paint have greatly reduced the incidence of dangerous lead levels in children, many children are still at risk for damaging lead exposure. Lead paint and the related dust and chips are the leading cause of high lead levels in U.S. children (Levin et al. 2008). Nontrivial sources of lead poisoning are contributed by lead-contaminated water, soil, and dust, although the condition of lead-based paint is a strong predictor of lead in house dust (Lanphear et al. 1998).

Other incidental sources of household lead exposure include the manufacture of stained glass and glazed pottery, remodeling of homes, toys or pottery containing lead-based paints (Mid-Atlantic Center for Children’s Health and the Environment 2003), certain calcium supplements including antacids and infant formula (Scelfo and Flegal 2000), and secondhand smoke (Mannino et al. 2003). Levin et al. (2008) document additional sources of lead exposure in eating utensils, breast milk, chocolate, candy, and other imported foods and related packaging.

Unfortunately, assessing the costs of removal of all lead hazards is difficult, so this analysis is restricted to the most common source of dangerous lead in children’s environments: lead-based paint. Although I posit an adjustment for this assumption in the final sections of this article, this restriction downwardly biases the costs estimates, inflating the return on investment.

### Lead paint in housing

Lead paint was used frequently in housing units until its ban in 1978; occupants of pre-ban houses are at a significantly greater risk for lead exposure. For these older housing units, the U.S. Department of Housing and Urban Development’s (2002) lead guidelines list several methods of safely controlling the lead hazard possibilities, including paint stripping, replacement, encapsulation, and enclosure. Jacobs et al. (2003) present a case study in which the costs of improper removal of lead-based paint were examined. They found the cost of decontamination after uncontrolled use of power sanders to be $218,320 for a single house, greatly exceeding the incremental costs of incorporating lead-safe work practices into repainting, a cost they estimated to be $1,200 for the individual homeowner [in 2006 U.S. dollars (USD)].

The President’s Task Force on Environmental Health Risks and Safety Risks to Children (2000) estimates the costs for two methods of controlling lead-based paint hazards. The first is lead hazard screening and interim controls, estimated to cost $1,200 per housing unit. The second method is inspection, risk assessment, and full abatement of lead paint, estimated to cost $10,800 per housing unit. Because of the variation in abatement requirements, regional differences in costs, condition of housing stock, and variation in the costs of adequate supervision and regulation of such work, the costs of lead hazard control can best be identified by a range rather than a precise estimate. Using the lower and upper bound values found in the President’s Task Force (2000), it is likely that the true cost lies in the range of $1,200–$10,800 per housing unit. This is line with the finding of Korfmacher (2003) that the national average cost of making housing lead-safe is $7,000 per unit.

According to the U.S. Department of Housing and Urban Development (2002), 38 million U.S. homes have lead paint, of which 24 million housing units were deemed to have lead hazards in 2000 (Jacobs et al. 2002). Four million of these homes have young children, and 1.2 million houses are at significant risk, with low-income families and children ≤ 6 years of age. Linearly extrapolating predicted reductions in units at risk of lead paint hazards from the President’s Task Force (2000), 1.02 million homes are at significant risk in 2006. Targeting these 1.02 million homes most in need and using the bounds on costs of $1,200–$10,800 per housing unit, the estimated cost lies between $1.2 billion and $11.0 billion.

## Benefits to Reduction

### Health care costs

High lead levels can cause multiple and irreversible health problems, which include learning disabilities, attention deficit–hyperactivity disorder (ADHD), mental retardation, growth stunting, seizures, coma, or, at high levels, death. Previous studies have identified damaging effects of lead on the nervous, hematopoietic, endocrine, and renal systems (Bernard 2003).

Treatment for low lead levels entails continuous monitoring of blood levels and prevention of further exposure, whereas higher lead levels require chemical chelation to leach lead from the body, an expensive, time-consuming, painful, and sometimes dangerous procedure. Kemper et al. (1998) have provided the most comprehensive assessment of health care costs. They estimate the cost for CDC’s prescribed medical interventions at each blood lead range.

Kemper et al. (1998) estimated costs of screening and treatment as follows: venipuncture ($8.57), capillary blood sampling ($4.29), lead assay ($23), risk assessment questionnaire ($2), nurse-only visit ($42), physician visit ($105), environmental investigation and hazard removal ($440), oral chelation ($332), and intravenous chelation ($2,418). These costs have been inflated to 2006 USD using the overall Consumer Price Index, an arguably conservative estimate of medical inflation because medical costs have increased at rates significantly higher than general inflation over the past decade. As children’s BLLs increase, so do their medical costs. Based on the assumptions of Kemper et al. (1998) and the CDC (2004) recommendations, it is possible to estimate the health costs per child given the levels of lead found in the population.

Although there is no BLL below which adverse health effects have not been observed (Bernard 2003; Binns et al. 2007; Brown 2007), the costs of medical diagnostics, prevention, and treatment for those with BLLs < 10 μg/dL are not included in this analysis because the medical costs of treating those below this CDC intervention level have not been fully assessed in the literature. To the extent that this omission is substantial, the medical benefits to lead hazard control are underestimated. This analysis also assumes that children who need treatment receive treatment immediately. If immediate treatment delays future health problems, and thus costs, then the medical benefits are again underestimated.

For children with levels ranging from 10 to 20 μg/dL, further diagnostic testing is required, necessitating venipuncture and a lead assay, followed by an additional nurse-only visit, for a total cost of $74 per child. For children with levels ranging from 20 to 45 μg/dL, the CDC (2004) recommends eight visits for diagnostic testing, including a nurse follow-up, and environmental investigation of the home in question, for a total cost of $1,027 per child. For children with BLLs of 45–70 μg/dL, the recommended regime includes all of the above, accompanied by oral chelation, for a total cost per child in the range of $1,335. For children with levels ≥ 70 μg/dL, oral chelation is replaced with intravenous chelation, for a total cost of $3,444 per child.

The estimated number of children affected in each group is a combination of two sets of data: pooled NHANES (2003–2006) and state child blood lead surveillance data from the National Center for Environmental Health (CDC 2007a). Given the relatively low level and nonrepresentative nature of state-level testing, the 39,526 children with BLLs > 10 μg/dL (as reported by the states) represent an absolute lower bound of prevalence. According to analysis of NHANES 2003–2006, 194,227 children have BLLs > 10 μg/dL. Because small sample sizes prevent accurate categorizing of children into each subgrouping of BLL, the upper bound is extrapolated by applying the ratio of confirmed cases in the CDC state-level surveillance data (CDC 2007a) to the numbers found in the NHANES and applying it to each subgroup. For example, because 39,526 is 20.35% of 194,227, the upper bound of children affected in the 10- to 15-μg/dL group is 24,554 confirmed cases divided by 20.35%, or 120,656 children. Table 2 reports the health care costs and incidence by BLL groupings. Summing across groups, the total cost of treatment is between $10.8 and $53.1 million.

The estimated range includes only the direct lead treatment costs for children ≤ 6 years of age. Lead poisoning causes negative health effects later in life, such as neurologic disorders, adult hypertension, heart disease, stroke, kidney malfunction, elevated blood pressure, and osteoporosis (Korrick et al. 2003; Latorre et al. 2003; Muntner et al. 2005). Many of these conditions are chronic illnesses that must be managed throughout an individual’s life course with either expensive pharmaceuticals or continual medical interventions. The biological effects of lead poisoning do not appear to affect all populations equally. Mexican-American and African-American populations possess a disproportionately strong relationship between elevated lead levels and hypertension, among other arterial diseases (Muntner et al. 2005).

### Social and behavioral costs

The most well-established area of research on the effects of BLLs on children and society centers around the relationship between high BLLs and cognitive and behavioral impairment. Even low levels of exposure appear to lower children’s IQ, which increases the need for enrollment in special education services, reduces the likelihood of high school and college graduation, lowers lifetime earnings (both through educational and IQ pathways), and greatly increases their propensity to engage in violent criminal activity. In this section I examine each of these factors in turn, assessing the evidence and determining the costs of lead exposure to the individual and society.

#### IQ and lifetime earnings

A variety of studies analyze the effects of high BLLs on intellectual function, most frequently quantified by IQ. Lanphear et al. (2005) have established a clear nonlinear, negative relationship between IQ and BLL based on pooled international data. The rate of IQ loss is greatest per unit blood lead < 10 μg/dL.

Data from NHANES (2003–2006) and state-level surveillance of lead poisoning (CDC 2007a) determine the number of children ≤ 6 years of age affected at each BLL ≥ 2 μg/dL (Table 3) . The average BLL for the 2- to 10-μg/dL group is based on the NHANES (2003–2006), the average BLL for the 10- to 20-μg/dL group is taken at the midpoint, assuming a uniform distribution of lead levels within the group, and the average BLL for the ≥ 20-μg/dL group is taken at 20 μg/dL. The small sample size does not allow for accurate estimates of average levels > 10 μg/dL; however, the assumption of the minimum is most conservative. Average IQ loss per 1 μg/dL is derived from the findings of Lanphear et al. (2005), assuming an even distribution of IQ loss within each BLL group.

Total IQ loss is computed for each BLL group, summed, and then multiplied by the estimated number of children affected. IQ loss from elevated BLLs falls between 9.3 and 13.1 million points. Although these losses have severe social and behavioral consequences, they also carry a significant financial burden of lost lifetime earnings.

Drawing from Salkever (1995), Schwartz (1994), and Nevin et al. (2008), I suggest that each IQ point loss represents a loss of $17,815 in present discounted value of lifetime earnings (in 2006 USD). Using the previously computed total IQ loss of 9.3–13.1 million points, net lifetime earnings loss is calculated to fall between $165 and $233 billion for all children ≤ 6 years of age in the 2006 cohort. This estimate includes the indirect effects of lower educational achievement and workforce participation in addition to the direct effect of lower hourly wages.

With every loss in lifetime earnings comes an associated loss in potential tax revenue for the government. Korfmacher (2003), using the methodology of Grosse et al. (2002), estimates that the state of New York is losing nearly $78 million in tax dollars each year because of lowered earnings from lead poisoning. If we perform the same exercise with a 15% marginal tax, lost tax revenue from lead poisoning is estimated to be $25–$35 billion for each cohort of lead-poisoned children.

#### Special education

Children with high lead levels are in need of special education because of their slower development, lower educational success, and related behavioral problems. Schwartz (1994) found that 20% of children with BLL > 25 μg/dL needed special education. He suggests that the needs of these children span an average of 3 years, requiring assistance from a reading teacher, psychologist, or other specialist. Korfmacher (2003) estimated that the average annual cost of special education is $14,317 per child (inflated to 2006 USD).

Based on the findings of Schwartz (1994), the 20% of children with BLLs > 25 μg/dL is estimated to fall between 693 and 3,404 children (using the same bounds analysis as described previously). Multiplying out these factors with the average cost per child for 3 years of special education, it costs an estimated $30–$146 million for each cohort of lead-poisoned children.

In addition to the relationship of reduced IQ on lifetime earnings and the additional investments required in special education, research indicates adverse effects of lead exposure directly on educational achievement and children’s readiness for school (Rothstein 2004). In addition, studies have found significant and negative effects of early and minimal lead blood exposure on statewide exam scores, in the same order of magnitude as the effect of poverty (Miranda et al. 2007).

Elevated BLLs are associated with an increased risk of not completing high school (Needleman 2004). Cohen et al. (1998) quantified the effects of dropping out of high school on lowered lifetime earnings and increased criminal activity. Although there may be a direct link between elevated lead levels and high school completion, this analysis chooses to avoid any potential double-counting and assumes that these effects are included indirectly in the earnings and criminal activity discussions. Excluding the nonmarket benefits of education (Haveman and Wolfe 1984) leads to an underestimate of the benefits of lead hazard control.

Research by Braun et al. (2006) has quantified the long-observed association between childhood lead exposure and development of ADHD. ADHD is a highly prevalent, lifelong psychiatric disorder that places children at an increased risk for conduct disorder, antisocial behavior, criminal activity, and drug abuse (Costello et al. 2003). Prevalence is estimated at 3–8% of children ≤ 15 years of age (CDC 2005). ADHD is managed through a combination of prescription drug therapy and counseling sessions for children and more severe adult cases. In addition to high lifelong treatment costs, ADHD also extracts significant productivity costs for parents of ADHD children. Work by Birnbaum (2005) finds that the parents of a child with ADHD collectively incur approximately $5 billion in work and productivity losses.

The total cost of lead-linked ADHD cases in the United States is found by computing the number of ADHD cases annually linked to early lead exposure, extracted from the study of Braun et al. (2006). Of the 1.8 million ADHD cases in children 4–15 years of age, 21.1%, or 290,000, are linked to BLLs > 2 μg/dL (Braun et al. 2006). Assuming average medical treatment costs per child of $565 for drug and counseling therapy and average parental work loss costs of $119 per child, lead exposure costs $267 million annually to individual families and society. Because the costs of medical treatment and work losses are likely to increase greatly with the severity of the condition, these estimates represent a conservative lower bound for the total costs of lead-linked ADHD cases.

#### Behavior and crime

Medical and economic research has established a connection between early childhood lead exposure and future criminal activity, especially of a violent nature. Bellinger et al. (1994) found that increased lead exposure correlates strongly with social and emotional dysfunction. Needleman et al. (1996) examined schoolchildren between the ages of 7 and 11 years who had a clinical diagnosis of lead poisoning at an early age and found worsening of behavior patterns as children with high BLLs aged. Needleman et al. (2002) indicated that adjudicated delinquents are four times more likely to have blood lead concentrations > 25 ppm than nondelinquent adolescents.

Recent work by Wright et al. (2008) examined a cohort of young adults from childhood and found a considerably higher and significant rate of arrest, particularly for violent crimes, among young adults who had elevated lead exposures at an early age. These clinical findings confirm broader research that links lead exposure to antisocial and destructive behavior, both in humans and animal subjects (Canfield et al. 2004; Denno 1990; Froehlich et al. 2007; Surkan and Zhang 2007).

Nevin (2000) finds that the variation in childhood gasoline lead exposure from 1941 to 1986 explains nearly 90% of the variation in violent crime rates from 1960 to 1998, and that lead paint explains 70% of the variation in murder rates from 1900 to 1960. Reyes (2002) takes the evidence of a relationship between lead poisoning and criminal behavior and estimates that the Clean Air Act (U.S. Environmental Protection Agency 2009) in the 1970s and 1980s accounts for one-third of the drop in crime throughout the 1990s.

Both clinical and econometric evidence suggest that lowered lead levels will lead to lower crime rates. The Federal Bureau of Investigation (2006) lists numbers of crimes per 100,000 residents, and the U.S. Bureau of Justice Statistics (2004) estimates their associated direct costs. Using Nevin’s (2006) estimate of the annual number of crimes that could have been averted with a 1-μg/dL reduction in the average preschool blood lead, the total direct costs of lead-linked crime can be computed.

A 1-μg/dL reduction in the average pre-school BLL results in 116,541 fewer burglaries, 2,499 fewer robberies, 53,905 fewer aggravated assaults, 4,186 fewer rapes, and 717 fewer murders (Table 4). The total direct cost of lead-linked crimes is approximately $1.8 billion, including direct victim costs, costs related to the criminal justice system through legal proceedings and incarceration, and lost earnings to both criminal and victim. An additional $11.6 billion is lost in indirect costs, which include psychological and physical damage necessitating medical treatment and preventive measures resulting from the criminal action. For this conservative analysis, I considered only the direct costs of each crime. Although these effects are for only a 1-μg/dL decrease, complete removal of lead hazards would have even larger effects.

The consequences of an antisocial and destructive pathology among lead-poisoned children are not isolated to criminal activity alone. Recent research has indicated that moderate levels of childhood lead exposure can greatly increase an individual’s propensity for risk-taking activities. For instance, Lane et al. (2008) found that BLLs > 20 μg/dL are strongly linked to repeat teenage pregnancies and cigarette smoking among low-income youth, both of which incur sizeable costs to individuals, families, and society.

## Discussion

To demonstrate the cost-effectiveness of lead hazard control, I summed and compared the total benefits and costs of childhood lead level reduction. The costs of lead hazard control range from $1.2 to $11.0 billion. The benefits to lead hazard control is the sum of the costs for medical treatment ($11–$53 billion), lost earnings ($165–$233 billion), tax revenue ($25–$35 billion), special education ($30–$146 million), lead-linked ADHD cases ($267 million), and criminal activity ($1.7 billion), for a total of $192–$270 billion. The net benefit of lead hazard control ranges from $181 to $269 billion, resulting in a return of $17–$221 for each dollar invested in lead hazard control (Table 5).

The estimate of the benefits of controlling lead hazards presented in this paper is still quite conservative. The absolute lower bound of lead prevalence > 10 μg/dL uses state-level confirmed cases and excludes many important and potentially substantial costs. These include health care later in life, neonatal mortality, benefits of lead hazard control on property value and energy savings, community improvement, lead paint litigation, indirect costs to criminal activity, and other intangible benefits. Similarly, this analysis calculates the benefit for one cohort of U.S. children, whereas the duration of lead hazard controls are likely to endure for ≥ 6 years (Wilson et al. 2006). Including future cohorts and assessing a full lifetime of costs would vastly increase the benefit to lead hazard control.

That said, the major source, lead-based paint, is by no means the only source of dangerous lead exposures among children. If a similar distribution of lead exposures or high and low BLLs are found from both lead-based paint and other types of lead hazards, a rough adjustment for other major sources of lead exposures on these benefits decreases the final benefit range by 30%, because lead-based paint represents about 70% of childhood exposure to lead (Levin et al. 2008). This leads to a net benefit ranging from $124 to $188 billion, resulting in a return of $12–$155 for each dollar invested in lead paint hazard control.

## Conclusions

Public health and housing policy has been slow to address these remaining lead poisoning risks, moving incrementally with targeted, more reactive policies. If the cost of proactive and universal lead hazard control is seen as prohibitive, the costs of inaction have proven to be significantly greater. For every dollar spent on controlling lead hazards, $17–$221 would be returned in health benefits, increased IQ, higher lifetime earnings, tax revenue, reduced spending on special education, and reduced criminal activity.

To put these results in perspective, it is useful to compare these net benefits to an intervention commonly understood as tremendously cost effective—that of vaccinations. Cost–benefit analyses show that vaccination against the most common childhood diseases delivers large returns on investment, saving between $5.30 and $16.50 in costs for every dollar spent on immunizations (Zhou et al. 2005). Given the high societal costs of inaction, lead hazard control appears to be well worth the expense as well.

## Figures and Tables

**Table 1:** Demographics of childhood lead poisoning (%).

Characteristic	BLL 2–10 μg/dL	Share of total population ≤ 6 years of age^a^
Children ≤ 6 years of age	24.7	100.0
Sex		
Male	53.6	51.1
Female	46.4	48.9
Race		
White, non-Hispanic	47.4	57.9
Black, non-Hispanic	23.6	13.7
Hispanic	24.6	21.1
Other	4.6	7.3
Income (% federal poverty line)		
Up to 200%	60.2	46.4
200–400%	22.8	29.2
≥ 400%	17.1	24.4

Author’s analysis of NHANES (2003–2006).

a Shares of population ≤ 6 years of age by race do not match ratios in other data because of differences in sampling and definitions.

**Table 2:** Health care costs (2006 USD).^a^

Blood lead level (μg/dL)	Cost of recommended medical action ($)	Lower bound of affected children (no.)	Upper bound of affected children (no.)^b^	Lower bound cost ($)	Upper bound cost ($)
10–15	74	24,554	120,656	1,816,996	8,928,552
15–20	74	8,185	40,220	605,690	2,976,305
20–45	1,207	6,347	31,189	7,660,829	37,644,611
45–70	1,335	376	1,848	501,960	2,466,585
> 70	3,444	64	314	220,416	1,083,104
All levels		39,526	194,227	10,805,891	53,099,158

a Kemper et al. (1998) provided estimates for the costs of recommended action (inflated to 2006 USD).

b The upper bound values are calculated assuming that CDC state-level surveillance confirmed cases represent 20.35% of estimates > 10 μg/dL derived from NHANES (2003–2006): 39,536 confirmed cases to 194,227 cases as estimated from NHANES (2003–2006).

**Table 3:** Lead and IQ.^a^

BLL (μg/dL)	Lower bound of affected children (no.)	Upper bound of affected children (no.)	Average BLL per BLL group (μg/dL)^b^	Average IQ point loss per μg/dL^c^	Lower bound IQ loss	Upper bound IQ loss
2–10	5,632,147	7,400,920	3.13	0.513	9,043,482	11,883,583
10–20	32,739	160,876^d^	~ 15	0.19	199,053	978,129
≥ 20	6,678	32,815^d^	~ 20	0.11	46,946	230,690
Totals					9,289,482	13,092,402

a Data for children with BLLs < 10 μg/dL are estimated from CDC NHANES 2003–2006. Data for children > 10 μg/dL are from state-level surveillance and assume uniform distribution of cases within each BLL group. Lower and upper bound for 2- to 10-μg/dL group represents 95% CIs for NHANES estimate.

b Average BLL calculated for 2–10 μg/dL using CDC NHANES 2003–2006, average BLL for 10–20 μg/dL taken as the midpoint, and average BLL for ≥ 20 μg/dL group uses the most conservative lower bound (the floor) for the mean.

c Data from Lanphear et al. (2005); assume uniform decreases within BLL groups.

d Values calculated assuming that CDC confirmed cases represent 20.35% of all cases, given that CDC confirmed cases represent 20.35% of NHANES estimates for those > 10 μg/dL.

**Table 4:** Lead and crime.

Crime	All crimes per 100,000 residents (no.)^a^	Lead-linked crimes per 100,000 residents (no.)^b^	Total lead linked crimes (no.)	Direct costs per crime ($)^c^	Total direct costs ($)^c^
Burglaries	1335.7	38.7	116,541	4,010	467,329,410
Robberies	213.7	0.83	2,499	22,871	57,154,379
Aggravated assaults	352.9	17.9	53,904	20,363	1,097,628,286
Rape	37.6	1.39	4,186	28,415	118,945,567
Murder	8.3	0.238	717	31,110	22,305,512
Totals			177,847		1,763,363,153

a Calculated using crime incidence data from the Federal Bureau of Investigation (2006).

b Data from Nevin (2006).

c Data from the Bureau of Justice Statistics (2004); inflated to 2006 USD.

**Table 5:** Total costs and benefits of lead control.

	Conservative estimate	Optimistic estimate
Total benefit from lead reduction	$192.38	$270.45
Total cost of lead control	$11.02	$1.22
Total net benefit	$181.37	$269.23
Cost–benefit	1–17	1–221

All costs and benefits are in billions of 1996 dollars.
